# Salt intake and blood pressure in Iranian children and adolescents: a population-based study

**DOI:** 10.1186/s12872-021-01876-z

**Published:** 2021-02-02

**Authors:** Mohammad Hassan Emamian, Hossein Ebrahimi, Hassan Hashemi, Akbar Fotouhi

**Affiliations:** 1 Ophthalmic Epidemiology Research Center, Shahroud University of Medical Sciences, Shahroud, Iran; 2 Randomized Controlled Trial Research Center, Shahroud University of Medical Sciences, Shahroud, Iran; 3 Noor Ophthalmology Research Center, Noor Eye Hospital, Tehran, Iran; 4 Department of Epidemiology and Biostatistics, School of Public Health, Tehran University of Medical Sciences, Tehran, Iran

**Keywords:** Children, Hypertension, Iran, Risk factor, Salt intake

## Abstract

**Background:**

Previous studies have reported a high prevalence of hypertension in Iranian students, especially in rural areas. The aim of this study was to investigate the daily intake of salt in students and its association with high blood pressure.

**Methods:**

A random sub-sample was selected from the participants of the second phase of Shahroud schoolchildren eye cohort study and then a random urine sample was tested for sodium, potassium and creatinine. Urine electrolyte esexcretion and daily salt intake were calculated by Tanaka et al.’s formula.

**Results:**

Among 1455 participants (including 230 participants from rural area and 472 girls), the mean age was 12.9 ± 1.7 year and the mean daily salt intake was 9.7 ± 2.6 g (95% CI 9.5–9.8). The mean salt consumption in rural areas [10.8 (95% CI 10.4–11.2)] was higher than urban areas [9.4 (95% CI 9.3–9.6)], in people with hypertension [10.8 (95% CI 10.3–11.3)] was more than people with normal blood pressure [9.4 (95% CI 9.3–9.6)], and in boys [9.8 (95% CI 9.7–10.0)] was more than girls [9.3 (95% CI 9.1–9.6)]. Higher age, BMI z-score, male sex and rural life, were associated with increased daily salt intake. Increased salt intake was associated with increased systolic and diastolic blood pressure.

**Conclusion:**

Daily salt intake in Iranian adolescents was about 2 times the recommended amount of the World Health Organization, was higher in rural areas and was associated with blood pressure. Reducing salt intake should be considered as an important intervention, especially in rural areas.

## Introduction

Globally 70% of the causes of death in 2019 were due to non-communicable diseases, which has been increasing over the past years. Ischemic heart disease is responsible for 16% of deaths and is the leading cause of death. It is estimated that 1.13 billion people worldwide have high blood pressure, and two-thirds of them live in low- and middle-income countries. High blood pressure is a major cause of premature death in the world and reducing its prevalence by 25% by 2025 compared to 2010, is a global target, recommended by World Health Organization. However, the prevalence of high blood pressure is increasing, especially in developing countries [3].

High systolic blood pressure (SBP) has the largest impact on the number of deaths in the whole country and all provinces of Iran. Non-optimal control of SBP has reduced life expectancy in Iranian men and women by 3.2 and 4.1 years, respectively [4]. However high blood pressure in Iranian children is not fully investigated and High blood pressure is common even in Iranian children and adolescents [5], but there are still not many studies in this field.

We recently have shown a high prevalence of hypertension in Iranian children aged 6–12 years old in a large population-based study [6]. In that study the prevalence of hypertension and prehypertension in rural area were 14.96 and 22.70% respectively. Surprisingly in that study, the prevalence of prehypertension in rural children was about two times of urban children and the prevalence of hypertension in the rural area was about four times of urban children [6].

Defining the causes of hypertension in children, especially in the rural area was very important in policymaking. Policymakers should rapidly recognize the risk factors and causes of this situation and develop and run appropriate intervention.

One hypothesis for the high prevalence of hypertension in rural children is high intake of sodium. Some researchers had investigated the daily salt intake even in children [7], however there was not sufficient evidence about difference of salt intake by residence place and at best of our knowledge, there was no study about sodium intake in Iranian children.

In a recent national study, daily salt intake in people over 25 in Iran was reported at 9.52 g/day [8]. This salt consumption which is about twice the standard of the World Health Organization [9], is almost similar to the United Kingdom (9.0) and the United States (10.1) [10], more than Australia (8.7) [11], less than the India (10.98) [12], Latin America (10.49) [13], Japan (13.1) [10] and China (15.1) [10].

By measurements of sodium excretion from urine, we could compare the nutritional habits of urban and rural children. This study aims to investigate daily salt intake in children and adolescents and its associated factors including age, sex, body mass index and residence place. Another aim of this study is to investigate the relationship between blood pressure and salt intake. The results of this study would be very important for policymaking and public health.

## Methods

This study is a part of the 2nd phase of Shahroud schoolchildren Eye Cohort Study (SSCECS). The methodology of SSCECS was reported previously [14]. In brief 5620 schoolchildren aged 6–12 years in Shahroud, northeast Iran, participated in the study in 2015. By considering classrooms as clusters, children selected by random cluster sampling in the urban area and to have larger sample in rural area and also giving health services to these children, all children in the rural area invited to the study. In the 2nd phase of SSCECS, all students who participated in the 1st phase (5620 students) were invited for an eye examination, anthropometry, blood pressure measurements and interviews in 2018. A subsample of these students was randomly selected for checking spot urine Na, K, and Cr excretion.

Although 24-h urine sample is the best choice for measuring daily salt intake and recent evidences indicate potential risk for misclassification at the individual level by using spot urine samples [15], currently it is approved that a spot urine sample can fairly be used for estimation of salt intake at a population level [16–20]. Previous studies showed that casual urine Na/K ratio is a good estimate for 24-h NaCl excretion and urinary Na/K ratio especially in population level [21–24]. Given the high sample size in this study and feasibility for using spot urine, we assessed spot urine sample instead of 24 h urine sample.

After a careful explanation about the objectives of the study and obtaining informed consent from children and their parents, a causal mid-stream random urine sample was taken into a disposable urine cup. Urine samples were not collected if female participants were in the first 5 days of menstruation. Samples were transported to laboratory and analyzed on the same day. Urine samples were discarded if urine specific gravity was less than 1.005. Urine samples were analyzed for Na, K and Cr by an automated electrolyte analyzer and Ion Selective Electrode method (i-Smart 30 Pro electrolyte analyzer, Korea).

Among the eight formula for estimating population daily salt intake from a spot urine sample, the Tanaka approach is one of the best and was approved as a correct method even in children and young adolescents [17, 19]. Recently it also confirmed as a good method for estimating daily salt intake from a spot urine sample in the Iranian population [25].

The daily Na and K excretion were calculated by the formulae, proposed by Tanaka et al. [24] as below:

1. Predicted value of 24 h Creatinine excretion (PRCr) (mg/day) =  − 2.04 × age + 14.89 × weight (kg) + 16.14 × height (cm) − 2244.45;
2. Estimated 24 h urine sodium excretion (mEq/day) = 21.98 × XNa^0.392^; where (XNa = (Na_spot_/Cr_mg/L_))*PRCr).
3. Estimated 24 h urine Potassium excretion (mEq/day) = 7.59 × XK^0.431^; where (XK = (K_spot_/Cr_mg/L_)*PRCr).

By considering that 1 g Na = 2.54 g NaCl and 1 mEq Na = 1 mmol Na, the daily salt intake was calculated using following formula: NaCl (g/24 h) = Na (mmol/24 h)*58.4/1000.

About 90% of salt intake is excreted into urine and the remaining is lost through sweating and feces [26]. Therefore, it was advised to divide the estimated daily NaCl excretion in the urine by 90% of the value to estimate correct daily salt intake. However, in this study age group with minimum activity and temperate climate of study location, the urinary sodium excretion can be considered approximately equal to sodium intake [26].

Height of the participants was measured using a wall-mounted stature meter with an accuracy of 0.1 cm. Students' weight was measured using a digital scale with an accuracy of 0.1 kg. Similar to our previous work [6], the diagnosis of hypertension was based on measurements of z-scores for children’s height, systolic blood pressure and diastolic blood pressure. Prehypertension was defined as 1.28 ≤ z < 1.645 and hypertension was defined as z-score ≥ 1.645, both in systolic or diastolic blood pressure.

The mean, standard deviation and 95% confidence intervals for urine Na, K, Na/K ratio and estimated salt intake were reported by children’s age, sex, residence place, and hypertension status. Any differences in the above means were assessed by t-test and ANOVA. Scheffe multiple-comparison tests were performed for hypertension groups, where ANOVA tests were significant. The association of daily salt intake and Na/K ratio with blood pressure, body mass index, age, sex, and residence place were investigated with multiple linear regression models. The effect of cluster sampling on standard errors and sampling weight in urban and rural areas were considered in calculation of confidence intervals. All analyses were done using statistical package Stata V11.0. *p* values less than 0.05 were considered as significant.

## Results

All participants in the first phase (5620 individuals) were followed, and finally 5292 students (94.2%) participated in the second phase of the study. A urine sample was taken for 1499 individuals. Sodium, potassium, and creatinine tests were performed on 1455 samples, and 44 samples were excluded from the analysis due to low volume or poor quality (low specific gravity) and outlier data (one case). The final analysis was performed on 1,455 students (including 230 participants from rural area and 472 girls). The mean age of participants in this study was 12.9 years with a standard deviation of 1.7 years.

The mean daily salt intake was 9.7 ± 2.6 g (95% CI 9.5–9.8). By adding 10% to the estimate of sodium loss through sweating and defecation, the mean daily salt intake was 10.6 ± 2.8 (95% CI 10.5–10.8). Table 1 shows more description of daily salt intake as well as daily sodium, potassium, creatinine, and sodium-to-potassium excretion ratio by sex, place of residence, and age groups. As can be seen in this table, the mean salt consumption in rural areas [10.8 (95% CI 10.4–11.2)] is higher than in urban areas [9.4 (95% CI 9.3–9.6)], in people with high blood pressure is higher than normal blood pressure [10.8 (95% CI 10.3–11.3) vs (9.4 (95% CI 9.3–9.6)], and in boys [9.8 (95% CI 9.7–10.0)] is more than girls [9.3 (95% CI 9.1–9.6)]. Salt consumption increased with increasing in age.

**Table 1:** The mean, standard deviation and 95% confidence intervals (in parentheses) of Sodium, Potassium, and daily salt intake by age, sex, residence place and hypertension status, Shahroud, Iran, 2018

Independent variables	n	Spot urine			Calculated in 24 h			
		Na (mmol/L)	K (mmol/L)	Cr (mg/dl)	Na (mmol/L)	K (mmol/L)	Na/K	NaCl (g/day)
Sex								
Male	983	217.6 ± 67.2 (213.3–221.8)	76.8 ± 32.9 (74.7–78.8)	126.1 ± 58.0 (122.4–129.7)	168.1 ± 44.1 (165.3–170.8)	44.2 ± 11.0 (43.6–44.9)	3.9 ± 0.9 (3.8–3.9)	9.8 ± 2.6 (9.7–10.0)
Female	472	218.4 ± 77.2 (211.4–225.3)	76.7 ± 32.4 (73.8–79.6)	136.2 ± 68.3 (130.0–142.4)	159.5 ± 44.1 (155.5–163.5)	41.9 ± 9.8 (41.0–42.8)	3.9 ± 0.8 (3.8–3.9)	9.3 ± 2.6 (9.1–9.6)
* p* value		0.838	0.975	0.003	< 0.001	< 0.001	0.780	< 0.001
Age (years)								
9	47	207.5 ± 68.3 (188.0–227.1)	74.9 ± 33.0 (65.5–84.3)	113.0 ± 49.4 (98.9–127.2)	132.1 ± 40.2 (120.6–143.6)	34.2 ± 10.6 (31.1–37.2)	4.0 ± 0.9 (3.7–4.2)	7.7 ± 2.3 (7.1–8.4)
10	228	207.0 ± 70.0 (197.9–216.1)	74.9 ± 31.4 (70.9–79.0)	106.9 ± 50.2 (100.4–113.4)	144.8 ± 39.3 (139.7–149.9)	38.5 ± 10.5 (37.1–39.8)	3.9 ± 0.8 (3.7–4.0)	8.5 ± 2.3 (8.2–8.8)
11	293	210.3 ± 72.8 (201.9–218.7)	83.1 ± 34.7 (79.1–87.0)	124.6 ± 62.4 (117.5–131.8)	154.1 ± 44.7 (148.9–159.2)	42.1 ± 10.0 (41.0–43.3)	3.7 ± 0.8 (3.6–3.8)	9.0 ± 2.6 (8.7–9.3)
12	298	222.4 ± 69.5 (214.5–230.3)	77.6 ± 30.7 (74.1–81.1)	123.4 ± 52.3 (117.5–129.4)	171.9 ± 41.3 (167.2–176.6)	45.3 ± 9.1 (44.3–46.3)	3.8 ± 0.8 (3.8–3.9)	10.0 ± 2.4 (9.8–10.3)
13	215	226.1 ± 66.7 (217.2–235.0)	76.0 ± 30.7 (71.9–80.1)	135.9 ± 55.2 (128.5–143.3)	175.3 ± 37.8 (170.3–180.4)	45.8 ± 9.4 (44.5–47.1)	3.9 ± 0.8 (3.8–4.0)	10.2 ± 2.2 (10.0–10.5)
14	239	220.2 ± 71.8 (211.1–229.3)	70.8 ± 31.7 (66.8–74.9)	144.7 ± 68.4 (136.1–153.4)	179.3 ± 44.4 (173.7–185.0)	45.8 ± 10.5 (44.4–47.1)	4.0 ± 0.9 (3.9–4.1)	10.5 ± 2.6 (10.1–10.8)
15	135	228.4 ± 70.5 (216.5–240.3)	76.4 ± 37.6 (70.1–82.8)	158.6 ± 77.8 (145.5–171.7)	180.2 ± 44.1 (172.7–187.6)	46.3 ± 12.4 (44.2–48.4)	4.0 ± 1.0 (3.8–4.2)	10.5 ± 2.6 (10.1–11.0)
* p* value		0.009	0.003	< 0.001	< 0.001	< 0.001	0.001	< 0.001
Residence place								
Urban	1225	209.9 ± 64.6 (206.3–213.5)	77.5 ± 33.2 (75.6–79.4)	131.6 ± 62.0 (128.1–135.1)	161.6 ± 42.1 (159.2–163.9)	43.3 ± 10.7 (42.7–43.9)	3.8 ± 0.8 (3.8–3.9)	9.4 ± 2.5 (9.3–9.6)
Rural	230	260.1 ± 84.8 (249.0–271.1)	72.7 ± 29.9 (68.8–76.6)	117.4 ± 58.9 (109.7–125.0)	185.1 ± 49.8 (178.6–191.6)	44.7 ± 10.3 (43.3–46.0)	4.2 ± 0.9 (4.1–4.3)	10.8 ± 2.9 (10.4–11.2)
* p* value		< 0.001	0.041	0.001	< 0.001	0.061	< 0.001	< 0.001
Blood pressure								
Normal	1182	209.1 ± 65.3 (205.4–212.9)	76.8 ± 32.6 (74.9–78.7)	130.6 ± 61.1 (127.1–134.1)	161.4 ± 42.0 (159.0–163.8)	43.1 ± 10.4 (42.5–43.7)	3.8 ± 0.8 (3.8–3.9)	9.4 ± 2.5 (9.3–9.6)
Pre HTN	144	263.5 ± 77.4 (250.8–276.1)	76.5 ± 31.5 (71.4–81.7)	122.4 ± 55.5 (113.4–131.5)	180.1 ± 47.2 (172.4–187.9)	43.9 ± 11.3 (42.0–45.7)	4.2 ± 1.0 (4.1–4.4)	10.5 ± 2.8 (10.1–11.0)
HTN	125	247.2 ± 82.1 (232.8–261.6)	76.6 ± 35.1 (70.5–82.7)	125.7 ± 73.3 (112.9–138.5)	185.0 ± 52.1 (175.8–194.1)	46.6 ± 11.9 (44.5–48.7)	4.1 ± 0.9 (3.9–4.2)	10.8 ± 3.0 (10.3–11.3)
* p* value		< 0.001	0.995	0.250	< 0.001	0.002	< 0.001	< 0.001
Total participants	1455	217.8 ± 70.6 (214.2–221.5)	76.7 ± 32.7 (75.1–78.4)	129.3 ± 61.7 (126.2–132.5)	165.3 ± 44.2 (163.0–167.6)	43.5 ± 10.6 (42.9–44.0)	3.9 ± 0.9 (3.8–3.9)	9.7 ± 2.6 (9.5–9.8)

Na, Sodium; K, Potassium; Cr, Creatinine; Na/K, Sodium to Potassium ratio; HTN, Hypertension

Multiple comparison tests in Table 2 show that respect to normal blood pressure group, urine excretion of Na, Na/K ratio and NaCL are higher in hypertension and pre-hypertension groups, while K does not differ between blood pressure groups. The mean differences between hypertension and pre-hypertension groups were not significant for all investigated urine electrolytes.

**Table 2:** The Scheffe multiple-comparison tests for mean differences of urine parameters by hypertension groups

Urine parameters	Hypertension groups	Normal blood pressure		Pre-hypertension	
		Mean difference	*p* value	Mean difference	*p* value
Spot urine Na (mmol/L)	Pre-hypertension	54.33	< 0.001		
	Hypertension	38.03	< 0.001	− 16.30	0.148
Calculated 24 h Na (mmol/L)	Pre-hypertension	18.76	< 0.001		
	Hypertension	23.58	< 0.001	4.83	0.663
Calculated 24 h K (mmol/L)	Pre-hypertension	0.76	0.716		
	Hypertension	3.51	0.002	2.75	0.104
Calculated 24 h Na/K	Pre-hypertension	0.40	< 0.001		
	Hypertension	0.24	< 0.001	− 0.16	0.600
Calculated 24 h NaCl (g/day)	Pre-hypertension	1.10	< 0.001		
	Hypertension	1.38	< 0.001	0.28	0.663

Table 3 shows the association between the independent variables with daily salt consumption, the sodium to potassium ratio in the random urine sample, and the daily sodium to potassium ratio of the estimated daily urine in the multiple linear regression models. Increasing age from 9 to 15 years, increasing BMI z-score, male gender and rural life were all associated with increased daily salt intake.

**Table 3:** The association of independent variables with daily salt intake and Na/K ratio in multiple linear regression models, Shahroud, Iran, 2018

Independent Variables	Estimated daily salt intake		Spot urine Na/K ratio		Estimated daily Na/K ratio	
	Coefficient (95% CI)	*p* value	Coefficient (95% CI)	*p* value	Coefficient (95% CI)	*p* value
Age (years)	0.49 (0.40, 0.57)	< 0.001	0.13 (0.06, 0.20)	< 0.001	0.04 (0.01, 0.07)	0.009
BMI z-score	0.60 (0.51, 0.69)	< 0.001	0.05 (− 0.02, 0.12)	0.138	− 0.004 (− 0.03, 0.03)	0.181
Sex (male/female)	0.58 (0.28, 0.89)	< 0.001	0.17 (− 0.04, 0.39)	0.108	0.06 (− 0.06, 0.16)	0.608
Residence place (rural/urban)	1.42 (0.96, 1.89)	< 0.001	0.94 (0.63, 1.25)	< 0.001	0.39 (0.201, 0.447)	< 0.001

Na, Sodium; K, Potassium; CI, Confidence intervals

**Fig. 1:**
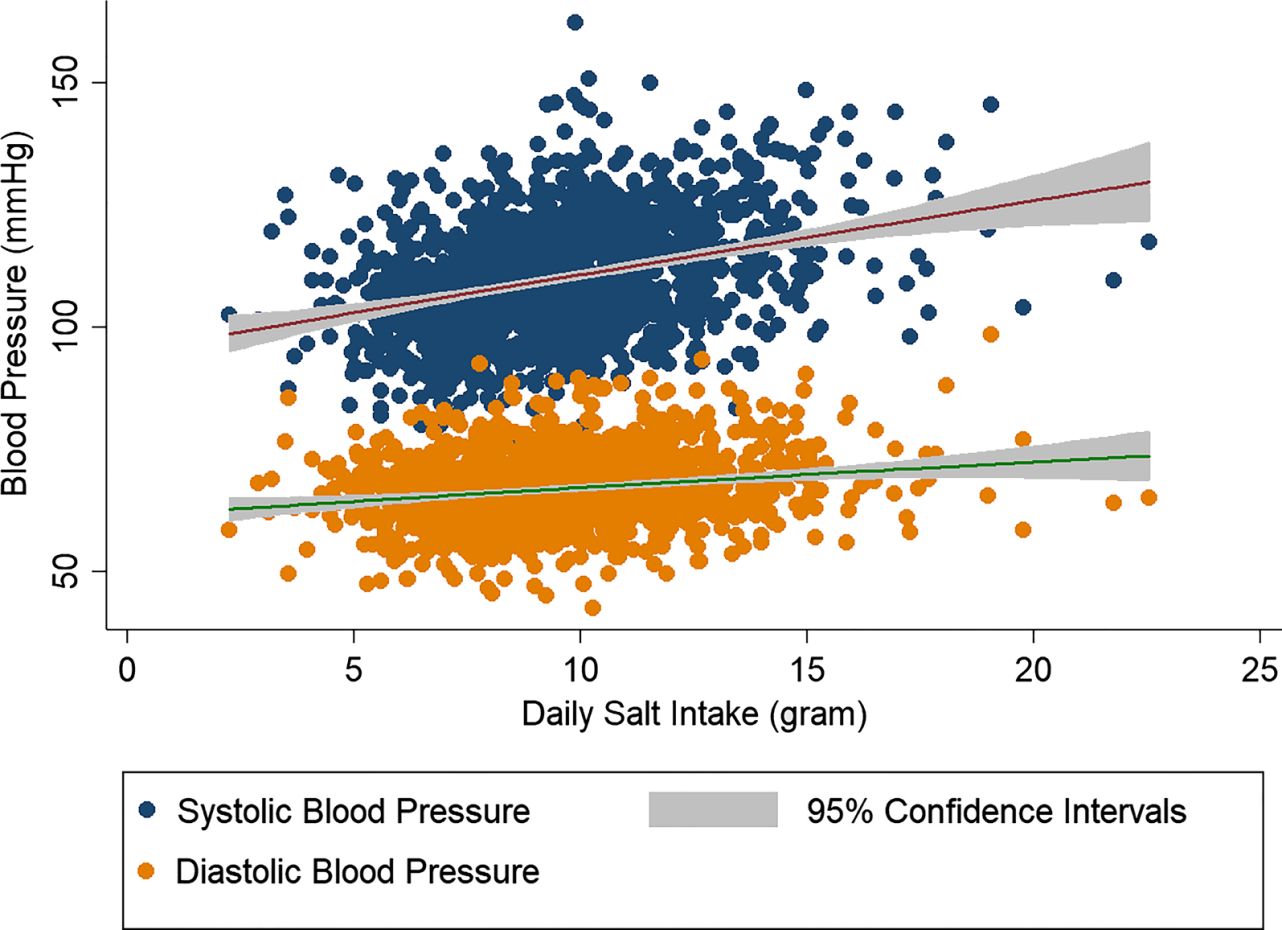
The correlation of systolic blood pressure and daily salt intake in 9–15 years old students by residence place, Shahroud, Iran, 2018

The association between systolic and diastolic blood pressure and salt consumption is shown in Fig. 1. Compared to diastolic blood pressure, increasing salt consumption is more strongly associated with increased systolic blood pressure. Table 4 also shows the association between systolic and diastolic blood pressure and daily salt intake in two multiple linear regression models, adjusted with age, gender, residence place and BMI-z score. Systolic and diastolic blood pressures increased by 1 mmHg for every 0.41 g and 0.18 g increase in daily salt consumption respectively. Although increased salt intake increases systolic and diastolic blood pressure, the place of residence has the greatest effect on the blood pressure (Table 4).

**Table 4:** The association of daily salt intake and other independent variables with systolic and diastolic blood pressure in multiple linear regression models, Shahroud, Iran, 2018

Independent variables	Systolic blood pressure		Diastolic blood pressure	
	Coefficient (95% CI)	*p* value	Coefficient (95% CI)	*p* value
Salt intake (g/day)	0.41 (0.17, 0.65)	0.001	0.18 (0.01, 0.35)	0.041
Age (years)	2.01 (1.58, 2.44)	< 0.001	0.77 (0.50, 1.05)	< 0.001
BMI z-score	2.79 (2.39, 3.19)	< 0.001	1.02 (0.72, 1.31)	< 0.001
Sex (male/female)	1.30 (0.07, 2.53)	0.038	− 1.07 (− 1.92, − 0.22)	0.014
Residence place (rural/urban)	8.20 (5.51, 10.90)	< 0.001	2.81 (1.49, 4.13)	< 0.001

**Fig. 2:**
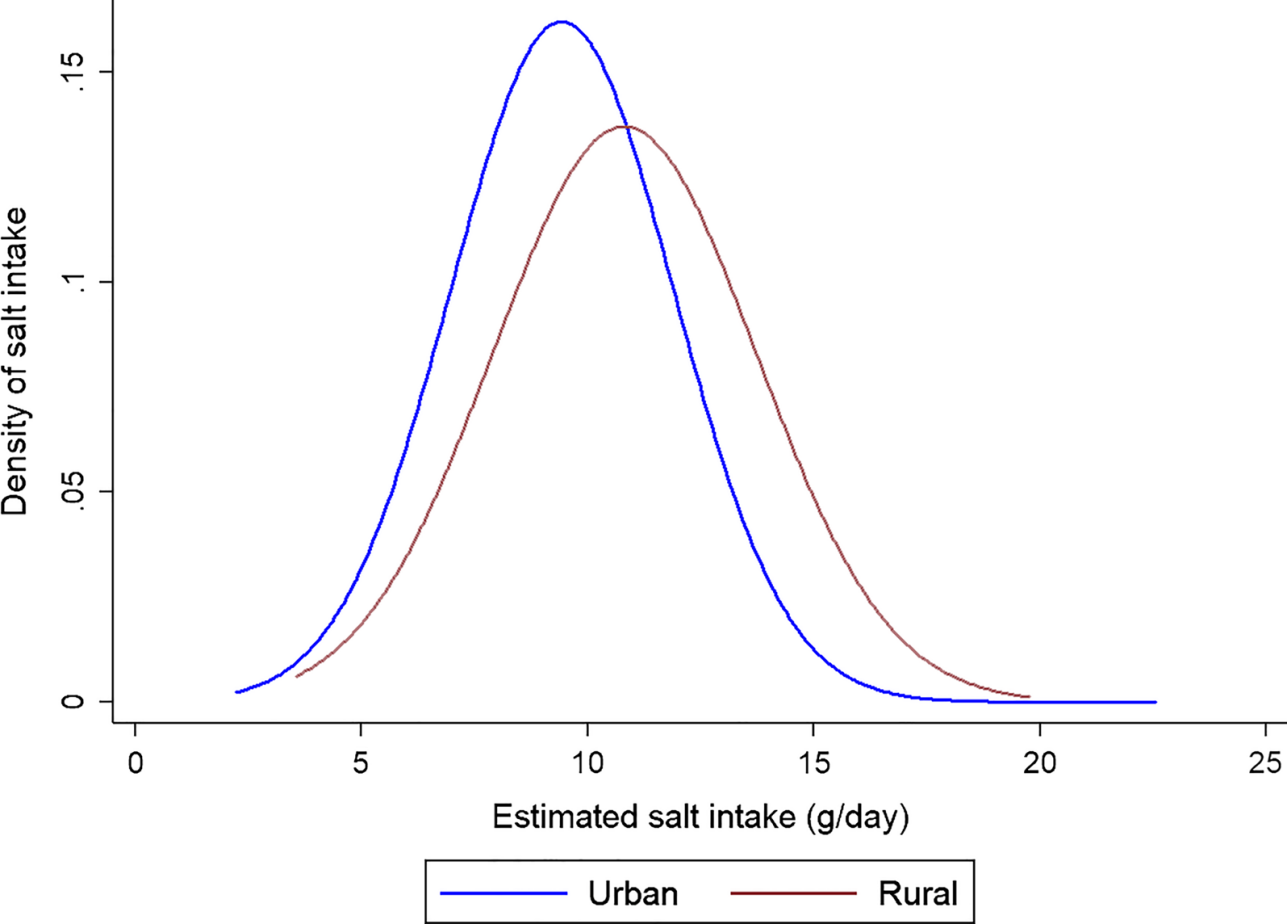
The distribution of daily salt intake in 9–15 years old students by residence place, Shahroud, Iran, 2018

**Fig. 3:**
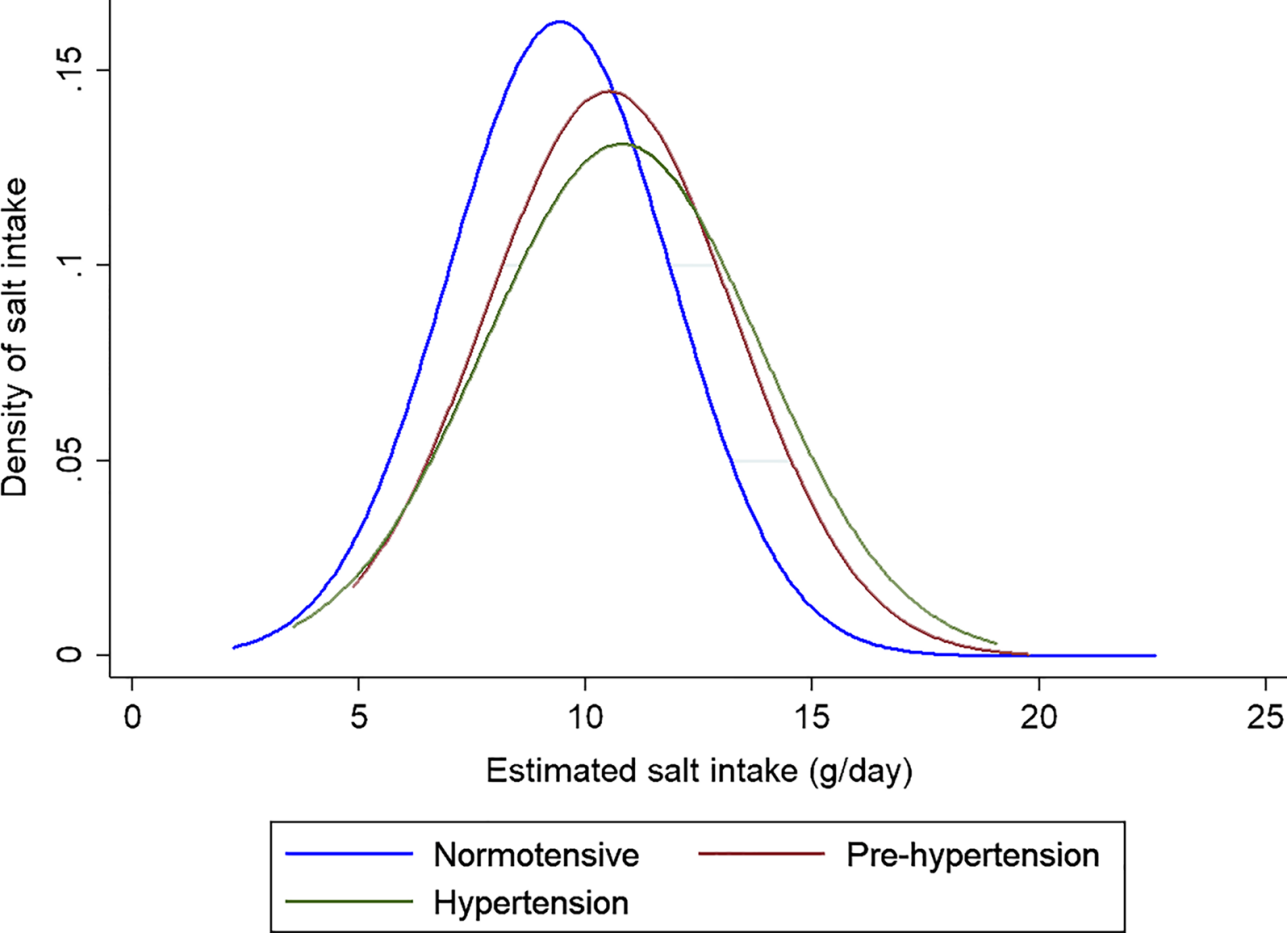
The distribution of daily salt intake in 9–15 years old students by hypertension groups, Shahroud, Iran, 2018

The distribution of daily salt intake according to the residence place is shown in Fig. 2 which shows that its distribution is almost normal and the rural students clearly have a shifted to the right distribution and receive more daily salt. Figure 3 shows the distribution of daily salt intake by different blood pressure groups. As expected, students with hypertension and pre-hypertension receive more daily salt.

## Discussion

This study investigated salt consumption in Iranian children for the first time in an area with a high prevalence of hypertension. The results of this study showed that the mean salt consumption in 9–15-year-old Iranian adolescents (9.7 g/d) is high and about twice the recommended level by the World Health Organization [9]. Salt consumption was higher in boys and rural areas. Systolic and diastolic blood pressure were also positively associated with salt intake. Although higher salt intake in rural areas partly justifies the increase in the prevalence of hypertension in rural adolescents, there are other factors that need further study.

Rezaei et al. [8] studied salt consumption in people over the age of 25 in different provinces of Iran and estimated it at 9.52 g per day [8]. The results of current study are very similar to the results of his national study and show that family eating habits determine the salt consumption of all family members [27, 28] and Iranian children have also high salt consumption. It should also be noted that in Rezaei et al. study [8], 10% was added to the value estimated by the Tanaka method, and if this happen in current study, the mean daily salt intake in adolescents (10.6 g/d) will be about 1 g higher than the mean national salt intake for individuals older than 25 years old. The estimation of Rezaei et al. for Semnan province, in which this study was conducted, is slightly higher than the estimate for whole country. Therefore, it can be said that two studies have had similar results.

To the best of our knowledge, another study in Iran did not examine salt intake in adolescents. In children aged 3–10 years in Isfahan, however, a study conducted in 2011–2012 on 241 children [29] showed that young Iranian children consumed large amounts of sodium. Also, the mean urinary sodium in Isfahan was 177.2 (somewhat similar to the results of the present study) and did not differ in sex and place of residence. The main sources of salt in that study were bread, cheese and snacks [29].

Other local studies have been conducted in Iran on the adult population and have had almost the same results as the present study. For example, a study among 20–74-year-olds people in Yazd estimated daily salt intake at 9.13 g [30]. In Isfahan, the mean salt consumption in people over 18 years of age was 9.5, 9.7, 9.6 and 10.2 g per day in 1998, 2001, 2007 and 2013, respectively [31] and it has been increasing from 2010 to 2014 [32]. Consumption of salt in Tehran in people over 19 years of age was 9.5 g per day [33]. The differences in the estimates of the above studies can be due to the type of method of measuring urinary sodium, the method of preparing the urine samples and the year of the study.

The results of the above studies show that prevention strategies for high salt consumption in Iran, which is said to have started in 2009 [34] with comprehensive policy at three levels of public information, food factories, and legislation [35] have not been very effective and needs to be seriously reconsidered.

Different levels of daily salt intake have been reported in different studies around the world. For example, it was 5.9 g/d in children 6–16 years old in an area in Switzerland [36], 5.7 g/d in Japanese children aged 9–11 years old [37], 7.2 g/d in Japanese children aged 6–12 years old [28], 6.5 g/d in Portuguese children aged 8–9 years old [38], 7.8 g/d in 7–11 years old Spanish children [7], 3.8, 4.7 and 7.6 g in children aged 5–6, 8–9 and 13–17 years old lived in south London respectively [39] and 7.4 and 6.7 in Italian boys and girls aged 6–18 years old [40]. These results show that salt consumption in Iranian adolescents is much higher than in other countries.

In the present study, the mean salt consumption in boys was higher than girls. This has been the case in other studies, such as in Portugal [38] or Italy [40] and even in adulthood [41]. It is believed that this difference reflects the different energy needs of boys and girls, so higher boys' need for energy leads to more food consumption and more salt consumption in them [38, 40].

According to the study results, the mean salt intake increased with increasing in age, BMI z-score and was higher in boys and rural area. In Portuguese children [38], salt intake was higher in boys and obese or overweight people and was similarly associated with systolic blood pressure but not with diastolic blood pressure. In 18–69-year-olds people in Shandong, China; the mean daily urinary sodium excretion was 237.6 mmol and the daily salt intake was 13.9 g, and the mean salt intake was higher in rural men and women [41]. In the same area, daily salt intake was higher in obese and overweight people than in normal weight, and sodium excretion was higher in people with hypertension and was associated with systolic blood pressure [42]. However, in a study by Kelishadi et al. in the age group of 3–10 years, there was no association between sodium intake and urinary excretion of sodium, potassium with systolic and diastolic blood pressure, which could be due to the age group of this study and low sample size [29]. Some studies have linked the association between blood pressure and salt intake to body weight and waist circumference [43]. While in this study, even in the presence of BMI-z score, blood pressure was associated with salt intake. The differences in age group and the prevalence of high blood pressure in these studies could be the reason for different results. In general, studies have shown that reducing salt intake not only lowers blood pressure but also has beneficial effects on vascular function and viscoelastic properties of large arteries [44]. It should be noted that researchers have recently concluded that in examining the association between salt intake and blood pressure, 24-h urinary sodium is more appropriate than the estimated sodium from spot urine [45].

In this study, daily salt consumption in rural areas (10.8 g/d) was higher than in urban areas (9.4 g/d). This situation is similar to most other studies [40, 46]. However, daily salt intake in urban areas is also high, and this difference in salt consumption justifies part of the higher prevalence of hypertension and pre-hypertension in rural area of Shahroud [6], and probably other nutritional differences in urban and rural areas are the main reason for the difference of blood pressure distribution in urban and rural areas in Shahroud. The results of a study on 12–19-year-olds adolescents in Shiraz, Iran also showed that western nutrition habits including high consumption of soft drinks, sweets and desserts, salt, mayonnaise, tea and coffee, salty snacks, high-fat dairy, French fries, and red or processed meats, are associated with increased blood pressure [47]. Therefore, in addition to reducing salt consumption, other nutritional interventions [48] are also necessary to reduce blood pressure in this area. Higher salt consumption in rural areas may be due to higher bread consumption, so interventions that reduce amount of bread salt and also bread consumption could lead to a decrease in systolic blood pressure in the short term [49].

The daily potassium excretion in this study was 43.5 mmol, which was higher in boys, older students and with no difference in urban and rural areas. This is higher than in countries such as Indonesia [50]—with a daily potassium excretion of 16.4 in children aged 9–12—and China [51]—with a daily excretion of 25.2 in children aged 6–16 years. But it is almost at the level of Italian children aged 6- to 18-year-old [40]. Insufficient potassium intake has been observed in other young populations around the world, for example in the young American population groups [52], among English children aged 7–10 years [53] and among French children [54]. Low Potassium intake in children and adolescents may be due to early dietary experience during infancy [55].

Although in this study, the mean urinary potassium of students with hypertension was higher than that of the normotensive and prehypertensive groups, in the multiple linear regression model and in the presence of other variables such as age, sex and BMI z-score, this association was not significant. This result was similar to other studies in Italy [40] and Uruguay [56]. However, the results of other studies in adults [57–59] show an inverse association between urinary excretion of potassium and blood pressure. Therefore, it is difficult to evaluate the possible effect of adequate consumption of this electrolyte on blood pressure in the study population of this study, where the prevalence of hypertension is high, and further studies are needed.

High sample size, accurate sampling in urban and rural areas and standard laboratory tests are the main strengths of this study, which was performed for the first time in the adolescent age group in Iran. However, the use of a random urine sample, diagnosis of hypertension based on measurement of blood pressure in one occasion, and not examining the, diet, physical activity and sleep of the participants may be considered as limitations of this study.

## Conclusions

The daily salt intake in Iranian adolescents is about twice the level recommended by the World Health Organization. This amount is higher in rural areas and people with high blood pressure, and therefore one way to reduce high blood pressure in rural areas is serious educational and nutritional interventions to reduce salt consumption. This is an important message to policymakers. However, investigating other causes of high prevalence of hypertension in Shahroud as well as continuous monitoring of blood pressure and salt intake is recommended.

## Data Availability

The datasets used and/or analyzed during the current study are available from the corresponding author on reasonable request.

## Abbreviations

DBP: Diastolic blood pressure
BMI: Body mass index
SBP: Systolic blood pressure
CI: Confidence intervals
Na: Sodium
K: Potassium
Cr: Creatinine
ANOVA: Analysis of variances
